# Virulence profile of carbapenem-resistant *Klebsiella pneumoniae* strains by an *in vivo* model of *Galleria mellonella*

**DOI:** 10.1128/spectrum.02215-24

**Published:** 2025-01-13

**Authors:** María Guembe, Rama Hafian, Marta Díaz-Navarro, Andrés Visedo, Flavio De Maio, Fulvia Pimpinelli, Ilaria Cavallo, Mauro Truglio, Francesca Sivori, Enea Gino Di Domenico

**Affiliations:** 1 Clinical Microbiology and Infectious Diseases Department, Hospital General Universitario Gregorio Marañón 16483 , Madrid, Spain; 2 IiSGM, Madrid, Spain; 3 University of Alcala, Madrid, Spain; 4 Dipartimento di Scienze di Laboratorio e Infettivologiche, Fondazione Policlinico Universitario A. Gemelli IRCCS, Rome, Italy; 5 Microbiology and Virology, San Gallicano Dermatological Institute, IRCCS 159149 , Rome, Italy; Griffith University-Gold Coast Campus, Gold Coast, Australia

**Keywords:** *Klebsiella pneumoniae*, resistance, carbapenemases, *Galleria mellonella*, carbapenems, lethality, biomass, metabolic activity, siderophore production, virulence

## Abstract

*Klebsiella pneumoniae* is a significant healthcare-associated pathogen, notable for its diverse virulence and antibiotic resistance profiles. This study aimed to characterize the genotypic and phenotypic diversity of *K. pneumoniae* isolates and evaluate their virulence using the *Galleria mellonella* model. Biomass production, metabolic activity, capsule formation, and siderophore production were assessed in 27 *K*. *pneumoniae* isolates from hospital-associated infections. Lethality curves were generated using the *G. mellonella* model, with survival monitored hourly from 16 to 48 hours. The most common sequence types (ST) identified were the high-risk clones ST307 (*N* = 10), ST512 (*N* = 8), ST101 (*N* = 7), and ST661 (*N* = 2). These STs were associated with distinct K-locus, including KL102, KL107, KL17, and KL39. Most isolates belonged to the O2afg locus (*N* = 18), with the *K. pneumoniae* carbapenemase genotype detected in 96.3% of strains. None of the isolates were classified as hypervirulent. Phenotypically, ST661 exhibited the highest biomass production despite showing similar metabolic activity to other STs. A positive correlation was observed between biomass and siderophore production, while capsule production was inversely correlated with biomass. In the *G. mellonella* model, ST661 demonstrated the highest virulence, resulting in 100% mortality by 48 hours, compared to survival rates of 21.4% for ST101, 38.0% for ST307, and 31.2% for ST512. These findings underscore the pathogenic potential of ST661 isolates with enhanced biofilm production. The *G. mellonella* model may serve as an effective *in vivo* system for evaluating the virulence of emerging *K. pneumoniae* lineages.

**IMPORTANCE:**

We demonstrate that the *Galleria mellonella* model is a useful tool to analyze the virulence of carbapenem-resistant *Klebsiella pneumoniae* strains. Our findings highlight the pathogenicity of carbapenem-resistant *K pneumoniae* isolates, particularly the role of the ST661 that, despite being a rare lineage, harbors the blaVIM gene and is associated with high biofilm production and the highest mortality rates.

## INTRODUCTION

*Klebsiella pneumoniae* is a gram-negative, encapsulated bacillus that colonizes human mucosal surfaces and is commonly associated with hospital-acquired infections (1, 2). Virulence in *K. pneumoniae* is multifaceted and plays a central role in its pathogenicity. Key virulence factors described in *K. pneumoniae* include pili, iron transporters, lipopolysaccharide (LPS), and the capsule. The *rmpA* gene, located either on the chromosome or on a large virulence plasmid, regulates the synthesis of capsular polysaccharides, contributing to capsule formation (3). The capsule and LPS are essential for immune evasion; LPS helps *K. pneumoniae* avoid detection by toll-like receptor 4 within host cells (4). Iron acquisition is another essential virulence mechanism, as iron is critical for bacterial growth and replication. Siderophores, such as enterobactin, yersiniabactin, salmochelin, and aerobactin, play a fundamental role in virulence by facilitating iron absorption, which is crucial for the bacteria’s metabolic activities and survival (5). Additionally, the presence of Pk genes is vital in bacteremia. These genes encode colibactin, a toxic substance that enhances the virulence of the strains (1).

*K. pneumoniae* can also form biofilms, an aggregate of bacteria and extracellular matrix produced by the microorganisms, which generally contain proteins, polysaccharides, and DNA. In the case of *K. pneumoniae*, this biofilm is mainly formed on artificial surfaces such as catheters or tissues, potentially causing invasive infections. Biofilm formation is crucial for maintaining the virulence activity of the bacteria in the host, where proteins like FabZ and LpxC are involved in its formation, and the protein KpOmpA plays a role in pathogenicity. Mutations in these proteins can lead to an imbalance in the biofilm’s homeostasis (2).

Acquired resistance of *K. pneumoniae* mainly occurs against carbapenem antibiotics, emerging after the widespread use of penicillin as a treatment for these infections. Resistance to carbapenems occurs due to the synthesis of carbapenemase enzymes by the bacteria, which hydrolyze the beta-lactam ring of the antibiotic, inhibiting its function (6, 7). The most concerning type of resistance is the production of class A carbapenemases, such as *K. pneumoniae* carbapenemase (KPC, predominantly distributed in the Mediterranean region, endemic in countries like Italy), oxacillinase-48 (OXA-48, in Spain), New Delhi metallo-beta-lactamase (NDM-1, in India), Verona integron-encoded metallo-beta-lactamase (VIM, in Greece and the USA), and imipenemase (IMP, in Japan). Recently, new virulence forms of *K. pneumoniae* have been reported, termed hypervirulent strains (hvKp) (8). The main characteristic of these strains is that they cause severe disease in healthy individuals (9, 10). These strains are characterized by an overexpression of siderophores, particularly aerobactin, a protein associated with iron transport, and phenotypically, they are distinguished from classical strains by hypermucoviscosity. In hypervirulent strains, there is an excess production of polysaccharides that form the capsule, as well as an overproduction of K and O antigen types, which are related to the protection of the bacteria from the environment and the formation of sugars in the lipopolysaccharide, respectively (11).

The growing prevalence of MDR and hypervirulent *K. pneumoniae* (hvKP) strains has led to global concerns about treatment limitations. The World Health Organization has highlighted the urgent need for research into resistance mechanisms and alternative treatment strategies, especially for pathogens where last-line antibiotics are losing efficacy (12). Understanding the relationship between virulence factors and antibiotic resistance in *K. pneumoniae* is essential for developing targeted interventions. Therefore, we aimed to study 27 *K*. *pneumoniae* strains according to different virulence factors to better clarify their pathogenicity based on a *Galleria mellonella* model, as this *in vivo* model has proven highly beneficial for determining the survival profiles of the strains without the necessity of employing vertebrate animal experimentation (13–18).

## MATERIALS AND METHODS

We analyzed 27 clinical strains of carbapenem-resistant *K. pneumoniae* (CRKP) isolated from hospital-related infections from the Microbial Strain Collection of the Microbiology and Virology, San Gallicano Dermatological Institute, Istituti Fisioterapici Ospitalieri, between January 2020 and March 2022. All clinical and microbiological data were recorded in a dedicated electronic database. The Central Ethics Committee I.R.C.C.S. Lazio approved this study (Prot. 5179—18.04.2023, N. 1860/23). All procedures and methods were performed according to the guidelines of the Ethics Committee and under local laws and regulations.

### Microbiological diagnosis and strain collection

*K. pneumoniae* exhibiting resistance to carbapenems were collected from patients with hospital-acquired infections (19). Cultures were plated on McConkey agar plates (Becton Dickinson, Heidelberg, Germany) and blood agar plates (Becton Dickinson, Heidelberg, Germany). Bacterial identification was performed using matrix-assisted laser desorption/ionization time of flight mass spectrometry (MALDI-TOF MS) (Bruker Daltonik, Bremen, Germany). Subsequently, 16S rRNA gene sequencing confirmed the identification (20). The antimicrobial susceptibility was assessed by the BD PhoenixTM automated microbiology system (Becton Dickinson Diagnostic Systems, Sparks, MD, USA). Susceptibility for colistin was determined by the broth microdilution test (Thermo Scientific, Massachusetts, USA). Results were interpreted according to the European Committee on Antimicrobial Susceptibility Testing (EUCAST) clinical breakpoints (http://www.eucast.org/clinical_breakpoints). The presence of blaKPC, blaVIM, blaOXA-48, blaIMP,and blaNDM genes was determined by the Cepheid Xpert Carba-R assay and the GeneXpert device (Cepheid, Sunnyvale, USA) (21).

### Whole-genome analysis

Genomic DNA for whole-genome sequencing (WGS) was extracted using the QIAsymphony DSP Virus/Pathogen Kits (Qiagen, Hilden, Germany) following the manufacturer’s protocols. High-quality DNA reads were processed using FastP v0.23.4 for trimming and assembled with SPAdes v3.15.5 (22, 23). The assembled genomes were annotated using Prokka v1.14.6. For pan-genome analysis, Roary v3.13.0 was utilized (24). Antibiotic resistance genes were predicted using the Comprehensive Antibiotic Resistance Database v3.2.8 and the Resistance Gene Identifier tool, with predictions restricted to “perfect” and “strict” matches against high-quality reference sequences, applying a 97% identity cutoff (25). Virulence factors were identified by performing a Blastn search against the Virulence Factor Database, considering hits with ≥80% coverage and ≥90% identity (26). Sequence typing of *K. pneumoniae* isolates was conducted by querying the PubMLST database. Kaptive v0.7.3 was employed to identify *K. pneumoniae* capsule (K-locus) and lipopolysaccharide (O-locus) loci (27).

### Virulence

For the determination of lethality curves as a virulence factor of *K. pneumoniae*, an *in vivo* model with *G. mellonella* larvae was used (ZooPinto S.L, Madrid, Spain). Bacterial suspensions of 10^5^ colony forming units ([CFU]/mL) were prepared using a turbidimeter, 0.1 µL of which was inoculated into the last left worm proleg, and larvae were incubated at 37°C (negative controls were only inoculated with phosphate-buffered saline [PBS]). Ten larvae were inoculated and monitored hourly for each strain from a 16-hour window until 48 hours. We classified as virulent, those strains that reached 100% lethality rate at the end of the 48-hour monitoring time. We decided to use 48-hour monitoring based on previous experiments in which we made the analysis based on two different criteria: >50% death at 16 h and 100% death at 48 h. As both were very similar, we decided to use criterion 2, as metabolic activity was lower in the virulent strains classified by this criterion, which correlates with pathogenesis (Fig. S1).

### Biofilm production

In parallel, using a static *in vitro* model, we quantified biomass and metabolic activity by 0.1% crystal violet (CV) and 0.5 mg/mL tetrazolium salt (XTT) assays, respectively. After 24 hours of incubation of the strains on agar plates for both experiments, they were inoculated into BHI (brain-heart infusion) and incubated on an orbital shaker at 150 rpm for 24 hours. After that, three centrifugation programs followed by three washes with PBS were performed. Once the centrifugations were completed, we prepared bacterial suspensions at 0.5 McFarland, and 100 µL of each suspension was added to a 96-well plate and left to incubate in the oven at 37°C for 24 hours. As a negative control, only BHI broth was added to the plate. After the incubation, the medium was gently aspirated to remove the non-adherent bacteria, and the plate was washed three times with 100 µL of PBS and allowed to dry.

For the CV assay used for biofilm biomass quantification, 125 µL of methanol was added to the wells and incubated for 15 min at room temperature. Then, methanol was removed, and 100 µL of CV was added, followed by a 10 min incubation at room temperature. The plate was then washed with sterile water and allowed to dry. Finally, 125 µL of 30% acetic acid was added and incubated for 10 min. The 125 µL of solubilized CV was transferred to a new plate, and the absorbance was read at 550 nm using a spectrophotometer.

To quantify the metabolic activity of the biofilm, 100 µL of XTT with menadione (at a ratio of 1 µL of menadione per 10 mL of XTT) was added to the wells, and the plate was incubated for 2 hours at 37°C protected from light. After the incubation period, the medium was transferred to a new plate, and the absorbance was read at 492 nm using a spectrophotometer.

The absorbance values obtained from the mean of the triplicates for each sample were recorded, subtracting the blank (negative control) for subsequent analysis in both assays.

### Capsule production

Capsule production was measured as previously described (28). One milliliter of overnight culture in a 1.5 mL microtube was centrifuged for 2 min at 7,000 relative centrifugal force (rcf). The supernatant was removed, and the pellet was resuspended in 1  mL of PBS. Subsequently, 875 µL of PBS-resuspended bacteria were mixed with 125  µL of Ludox LS colloidal silica (30% [wt/wt] suspension in water). This mix was then centrifuged for 30 min at 12,000 rcf, and the distance of the center of the band from the bottom of the microtube was measured. The measurements were taken in biological triplicates and statistically evaluated by calculating the standard deviation from the mean values for biological replicates.

### Quantitative measurement of siderophore

For each bacterial culture, 500 µL of broth was inoculated with diluted starter cultures adjusted to a final concentration of approximately 1 × 10^5^ CFU/mL in a 1.5 mL centrifuge tube. A control tube (uninoculated broth) was also included. After incubation at 37°C under static conditions, bacterial cultures were centrifuged, cell pellets were discarded, and the supernatant was used to estimate siderophore. Subsequently, 100 µL of supernatant of each bacterial culture was added in separate microplate wells, followed by 100 µL of chrome azurol S (CAS) reagent, prepared as previously described (29, 30). After 20 min, the absorbance of each well was measured spectrophotometrically at 630 nm using the Multiskan SkyHigh (Thermo Fisher Scientific, USA). Siderophore production was measured in percent siderophore unit (PSU), which was calculated according to the following formula (31).

Siderophore production (PSU %) = ([Ar − As]/Ar) × 100

Where Ar = absorbance of reference (CAS solution and uninoculated broth) and As = absorbance of sample (CAS solution and cell-free supernatant of sample).

### Statistical analysis

Values are expressed as the median interquartile range (IQR) for continuous variables and as percentages, with a 95% confidence interval, when applicable, for categorical variables. Categorical variables were evaluated using the *χ*^2^ test or a two-tailed Fisher exact test. Statistical significance was set at *P* < 0.05 (two-tailed). Statistical analysis was performed using IBM SPSS Statistics for Windows, Version 21.0 (IBM Corp, Armonk, New York, USA).

## RESULTS

The isolates were classified into four distinct sequence types (STs), represented by the high-risk clones ST307 (*N* = 10), followed by ST512 (*N* = 8), ST101 (*N* = 7), and ST661 (*N* = 2) (Table 1). These isolates were further categorized into four K-locus groups corresponding to their respective STs. The most common capsule types observed were KL102 (*N* = 10), KL107 (*N* = 8), KL17 (*N* = 7), and KL39 (*N* = 2). In terms of O-locus, the majority of isolates were identified as O2afg (*N* = 18), with O1ab (*N* = 7) and O3/O3a (*N* = 2) occurring less frequently. The predominant carbapenem-resistant genotype identified was KPC, which was present in 26/27 strains, while the remaining was the VIM genotype. To evaluate the presence of hvKP, the virulence genes *iroB*, *iucA*, *prmpA*, and *prmpA2* were screened. None of the strains tested positive for these genes, categorizing them as non-hypervirulent.

**TABLE 1:** Carbapenem-resistant *K. pneumoniae* isolates characterized in this study

ST	N° of strains	K-locus	O-locus	*bla* Gene
307	10	KL102	O2afg	*bla_KPC_*
101	7	KL17	O1ab	*bla_KPC_*
512	8	KL107	O2afg	*bla_KPC_*
661	2	KL39	O3/O3a	*bla_KPC_*
				*bla_VIM_*

**Fig 1:**
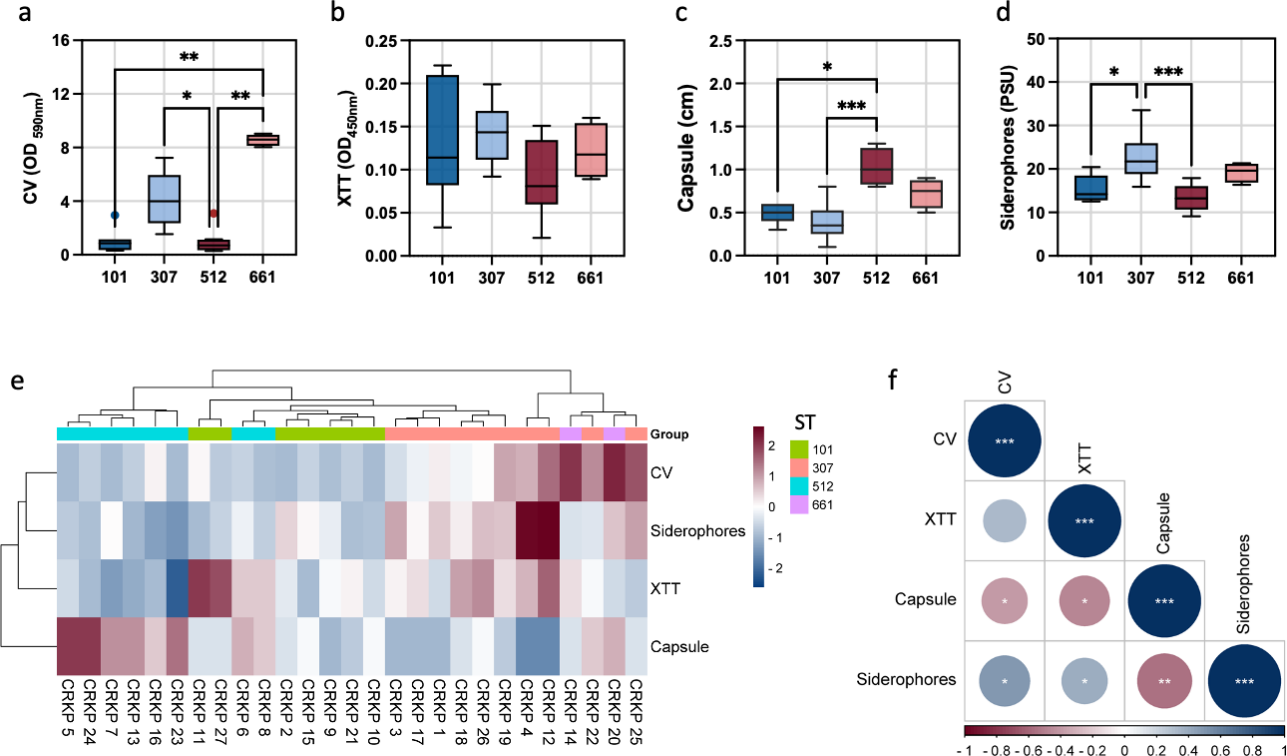
*K. pneumoniae* isolates distribution by virulence factors (a) CV, (b) XTT, (c) capsule production, and (d) siderophores expressed as PSU. Statistical differences were determined using the Kruscal-Wallis test. Post-hoc pairwise analyses were conducted with Dunn’s test, applying Bonferroni correction to adjust for multiple comparisons. (e) Cluster analysis based on ST. (f) Correlation analysis for CV, XTT, capsule, and siderophores production. Spearman correlation significance levels: *P < 0.05; **P < 0.01; ***P < 0.001.

To investigate virulence factor production of the isolates, biofilm formation, capsule production, and siderophores were measured. CV assay revealed that ST661 produced significantly higher biomass than ST101 (*P* = 0.0030) and ST 512 (*P* = 0.0013; Fig. 1a). No significant differences were observed in the metabolic activity within the biofilm, as measured by XTT, among the STs (Fig. 1b). Capsule production was significantly higher in ST512 than in ST101 (*P* = 0.0102) and ST307 (*P* = 0.0003; Fig. 1c). Siderophore production was significantly higher in ST307 than in ST101 (*P* = 0.0335) and ST512 (*P* = 0.0007; Fig. 1d). Cluster analysis showed that most of the isolates clustered together based on their ST (Fig. 1e).

Given the variability in the virulence phenotypes among the ST, it was questioned whether these phenotypes could be coregulated or if they might function independently. Direct correlations identified that biomass (CV) was positively correlated with siderophores production (*P* = 0.011). In turn, capsule production was negatively correlated with CV (*P* = 0.0430), XTT (*P* = 0.0106), and siderophores (*P* = 0.0024; Fig. 1f).

**Fig 2:**
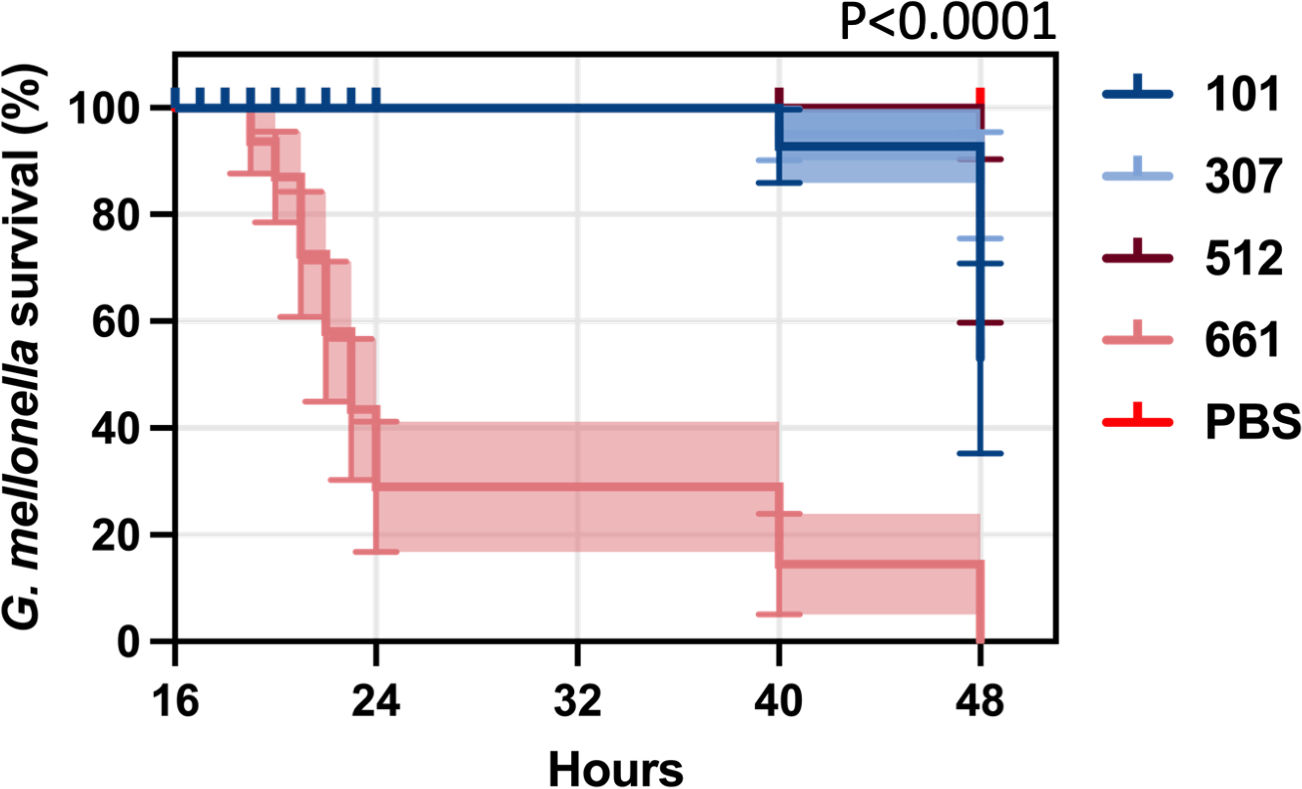
Survival percentage of *G. mellonella* larvae exposed to different *K. pneumoniae* isolates by ST. The survival of *G. mellonella* larvae was monitored over time following infection with various *K. pneumoniae* STs (ST101, ST307, ST512, and ST661) and a PBS control. The graph displays a time-dependent decrease in survival, with significant differences in pathogenicity observed between STs (*P* < 0.0001).

The *G. mellonella* larvae model was used to compare the *in vivo* pathogenicity of all CRKP isolates (Fig. 2). There was a significant difference in virulence among STs (*P* < 0.0001), with ST661 being associated with the highest mortality rates compared to the other STs (Fig. S2). Larvae survival rates at 48 hours were 21.4% for ST101, 38.0% for ST307, 31.2% for ST512, and notably, 0% for ST661.

## DISCUSSION

This study analyzed the genotypic and phenotypic profiles of 27 CRKP isolates from hospital-related infections. The predominant mechanism of carbapenem resistance was the expression of the *bla*_KPC_ gene, found in 96.3% of the isolates, with only one strain testing positive for *bla*_VIM_. These findings align with current literature, highlighting the widespread prevalence of *bla*_KPC_, the most common carbapenemase in CRKP isolates in Southern Europe (32, 33). WGS analysis identified four distinct STs—ST307, ST512, ST101, and ST661—highlighting the genetic diversity within the CRKP population. The high-risk clones ST307, ST512, and ST101 were the most prevalent lineages, consistent with their dominance in global surveillance studies. These STs have been identified as significant contributors to the burden of carbapenem-resistant infections, particularly in Southern European countries, including Italy, Romania, and Spain (32, 34, 35). In contrast to these dominant STs, ST661 is a relatively rare lineage in clinical settings. However, its emergence is of particular concern due to its involvement in a documented outbreak in the United Kingdom, where all clones harbored the *bla*_KPC_ gene. This outbreak was noteworthy for the covert dissemination of CRKP, highlighting multiple genetic modes of transmission among ST661 isolates (36). Moreover, one of the two ST661 isolates identified in our study was the only clone positive for the *bla*_VIM_ gene. This finding aligns with previous reports from Italy, where an ST661 clone carrying *bla*_VIM_ was identified in a surveillance rectal swab (37). Notably, ST661 is frequently detected in animals as a zoonotic opportunistic pathogen, with reports of its presence in cows, pigs, minks, and poultry (38–42). This clone may represent animal-adapted strains that humans are frequently exposed to through the food chain, with the potential to evolve into an epidemic or high-risk lineage (43). The detection of the *bla*_VIM_ gene in non-predominant *K. pneumoniae* clones, which have been shown to cause severe but sporadic outbreaks, may also indicate horizontal gene transfer events, potentially contributing to the spread of resistance genes across different lineages. This finding underscores the importance of monitoring for diverse carbapenemase genotypes to anticipate and manage emerging resistance patterns effectively. This is essential to mitigate the spread of multidrug-resistant clones that combine virulence factors beyond the classical hypervirulent traits, potentially becoming an epidemic or high-risk clone.

As detected by WGS, none of the isolates tested positive for classical hypervirulence genes such as *iroB*, *iucA*, *prmpA*, and *prmpA2*. This finding aligns with previous reports indicating that CRKP isolates are rarely classified as hypervirulent (44). Furthermore, a recent study demonstrated that multidrug-resistant HvKp exhibited comparable virulence in a mouse pneumonia model to non-hypervirulent multidrug-resistant clones and significantly higher virulence than hypervirulent strains alone (45). These observations suggest that the convergence of multidrug resistance and hypervirulence in *K. pneumoniae* may not necessarily correlate with increased pathogenicity in all models, particularly when compared to non-hypervirulent multidrug-resistant strains. This finding is consistent with emerging evidence that the genetic determinants of resistance and virulence can act independently or in complex interactions rather than simply compounding each other’s effects (46). While the sporadic convergence of classical hypervirulence markers in CRKP isolates is somewhat reassuring, the potential for these strains to cause severe infections remains a significant concern.

The phenotypic analysis conducted in this study revealed substantial variability in biofilm formation, capsule production, and siderophore levels among the different STs of CRKP isolates. ST661 exhibited the highest biomass production among the analyzed sequence types, a trait closely associated with enhanced antimicrobial tolerance and persistence, complicating infection management. This robust biofilm-forming capacity also provides a protective barrier against host immune defenses, facilitating chronic colonization and transmission within clinical settings (26, 27). Collectively, these attributes underscore the potential of ST661 as a high-risk clone, warranting further surveillance and investigation into its clinical impact. Although ST661 lacked classical hypervirulence genes, we cannot exclude the possibility that the increased virulence of these strains arises from a combination of other virulence factors not examined in this study, in addition to biofilm formation. This underscores the need for further comprehensive analyses to elucidate the pathogenic potential of ST661 fully. Capsule production was highest in ST512, while siderophore production peaked in ST307, underscoring the diversity in pathogenic strategies among these STs. The phenotypic analysis of our isolates revealed a positive correlation between biofilm formation and siderophore levels, along with an inverse correlation with capsule production. This confirms a complex regulatory interplay among virulence factors, consistent with findings from previous studies (47). Notably, other studies have shown that CRKP strains may develop pathogenicity through substantial changes in capsular polysaccharides, which can lead to two opposing *in vivo* evolutionary strategies during host infection, one involving increased host-cell adhesion and biofilm formation and the other enhancing virulence through elevated capsule production. These findings highlight the adaptive versatility of *K. pneumoniae* and the potential trade-offs between biofilm formation and other virulence factors in response to selective pressures within the host environment (21, 48).

Our analysis showed that more virulent strains in *G. mellonella* exhibited higher biofilm production, consistent with previous studies linking this trait to increased mortality in oncological patients (21). Interestingly, while these virulent strains had higher biomass, they also showed lower metabolic activity, as measured by XTT. This finding aligns with the understanding that biofilm-associated bacteria typically have reduced metabolic activity, making them more resistant to antibiotics, which require active bacterial processes to be effective (12).

Contrary to what might be expected, our study found that the less virulent ST307 in the *G. mellonella* model produced more siderophores than their more virulent counterparts. This suggests that siderophore production may not be considered a primary virulence determinant in these strains. Previous research has established that siderophores enhance bacterial virulence by facilitating iron acquisition (28). Still, our data suggest that *K. pneumoniae* can maintain high virulence through other mechanisms, even with reduced siderophore production (30).

The *G. mellonella* model revealed significant differences in virulence among the STs. ST661 exhibited the highest lethality, resulting in 100% mortality by 48 hours. This is particularly notable given the absence of traditional hypervirulence markers, indicating that ST661 may possess alternative virulence mechanisms that warrant further investigation. The distinct pathogenic potential of ST661, particularly its ability to form biofilms, highlights its potential role in severe clinical outcomes.

Regarding the use of the *G. mellonella* model, as demonstrated in other studies, it has proven highly beneficial for determining the survival profiles of the strains under investigation without employing vertebrate animal experimentation. Through this alternative method, it is conceivable that future research may diminish the reliance on higher-order mammalian animals, such as mice and rats while yielding comparably reliable outcomes. This model boasts numerous advantages, including the substantial similarity of the larval immune system to that of humans, possessing an optimal growth temperature closely akin to humans, and the cost-effectiveness associated with larvae experimentation (7–12).

In the literature, most related studies demonstrate a correlation and effectiveness with the aforementioned *in vivo* model, as a similar or identical inoculum dose is administered to the larvae. However, when lower inoculum doses are administered, this method loses effectiveness, and it becomes challenging to discriminate between virulent and non-virulent strains, as observed in the study conducted by Russo et al. Their analysis showed no differences between classical and virulent strains when administering lower bacterial inoculum doses. Nonetheless, as depicted in their results, differentiation between virulent and classical strains became feasible when higher infective doses were used (e.g., 10^5^ CFU/mL, as commonly performed in most related studies). Hence, it is crucial to consider the infective dose administered to the larvae to extrapolate data accurately and conduct comparisons and analyses that yield reliable results (23).

The limitations of our study, including the arbitrary selection of a 48-hour monitoring period, must be acknowledged. Moreover, as we only tested 27 strains, the statistical power of the analysis may be limited. Previous studies using the *G. mellonella* model have employed varying timeframes, ranging from 24 to 144 hours, which could influence the interpretation of virulence data (49). Additionally, while our isolates are multi-drug-resistant, the lack of traditional hypervirulence markers raises questions about the classification criteria for hypervirulence in *K. pneumoniae*. Whole-genome sequencing and further phenotypic assays are necessary to uncover potential virulence determinants that conventional markers may not capture.

### Conclusions

Our findings highlight the pathogenicity of CRKP isolates, particularly the role of the ST661, which, despite lacking traditional hypervirulence markers, demonstrates high virulence *in vivo*. Although ST661 is a rare lineage, it harbors the *bla*_VIM_ gene and is associated with high biofilm production and the highest mortality rates. The study underscores the need for ongoing surveillance and research to uncover the mechanisms driving virulence and resistance in *K. pneumoniae*, particularly in emerging high-risk clones.

## Data Availability

The data for this study have been deposited in the European Nucleotide Archive (ENA) at EMBL-EBI under accession number PRJEB82239.

## SUPPLEMENTAL MATERIAL

Fig. S1 spectrum.02215-24-s0001.docx Metabolic activity of *K. pneumoniae* strains according to virulence classification analyzed by 2 different criteria.

Fig. S2 spectrum.02215-24-s0002.docx Comparison of *Galleria mellonella* mortality rates between each sequence type isolates.

ASM does not own the copyrights to Supplemental Material that may be linked to, or accessed through, an article. The authors have granted ASM a non-exclusive, world-wide license to publish the Supplemental Material files. Please contact the corresponding author directly for reuse.
