# Supplementary material for: Virulence profile of carbapenem-resistant Klebsiella pneumoniae strains by an in vivo model of Galleria mellonella
Source: Microbiol Spectr. 2025 Jan 13;13(2):e02215-24. doi: 10.1128/spectrum.02215-24 (PMC11792541; doi:10.1128/spectrum.02215-24)
Supplement: Fig. S1 — Metabolic activity of K. pneumoniae strains according to virulence classification analyzed by 2 different criteria. [file spectrum.02215-24-s0001.docx]

**
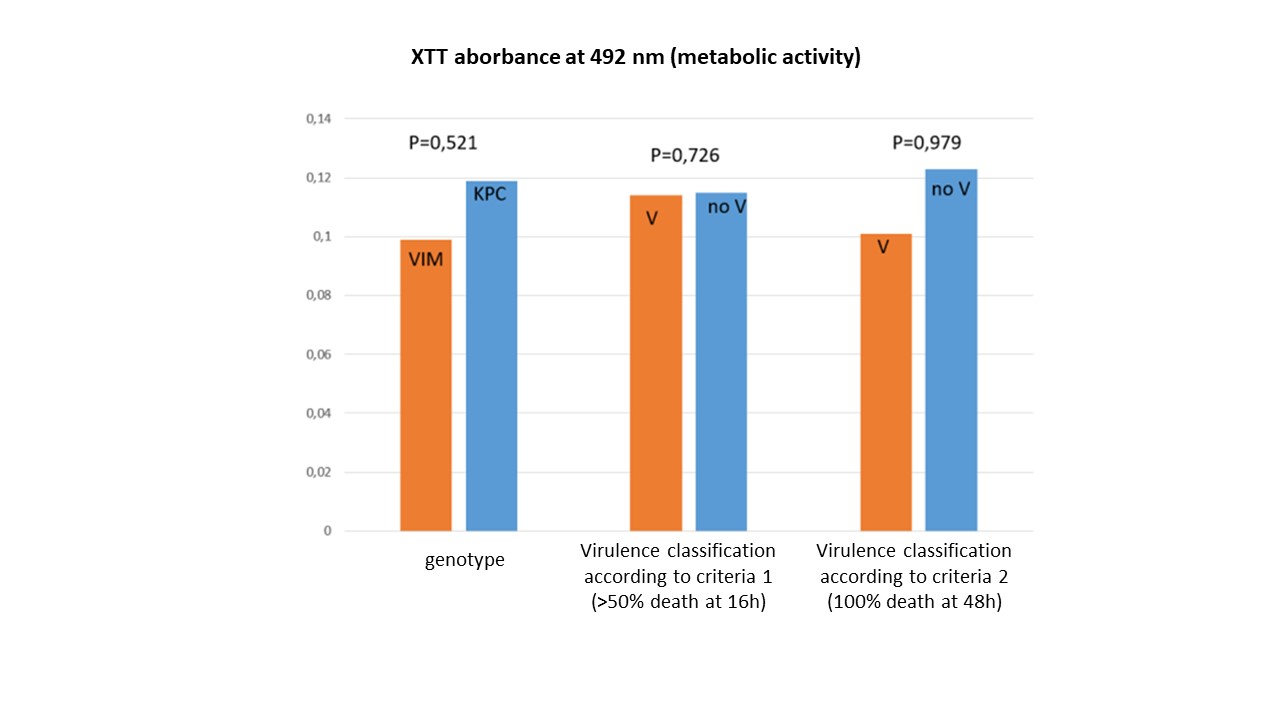
Supplemental material 1. Metabolic activity of *K. pneumoniae* strains according to virulence classification analysed by 2 different criteria**

**V**, virulent; **no V**; non-virulent; **KPC**, *Klebsiella pneumoniae* carbapenemase; **VIM**, Verona integron-encoded metallo-beta-lactamase.
