# Supplementary material for: Virulence profile of carbapenem-resistant Klebsiella pneumoniae strains by an in vivo model of Galleria mellonella
Source: Microbiol Spectr. 2025 Jan 13;13(2):e02215-24. doi: 10.1128/spectrum.02215-24 (PMC11792541; doi:10.1128/spectrum.02215-24)
Supplement: Fig. S2 — Comparison of Galleria mellonella mortality rates between each sequence type isolates. [file spectrum.02215-24-s0002.docx]

**Supplemental material 2.
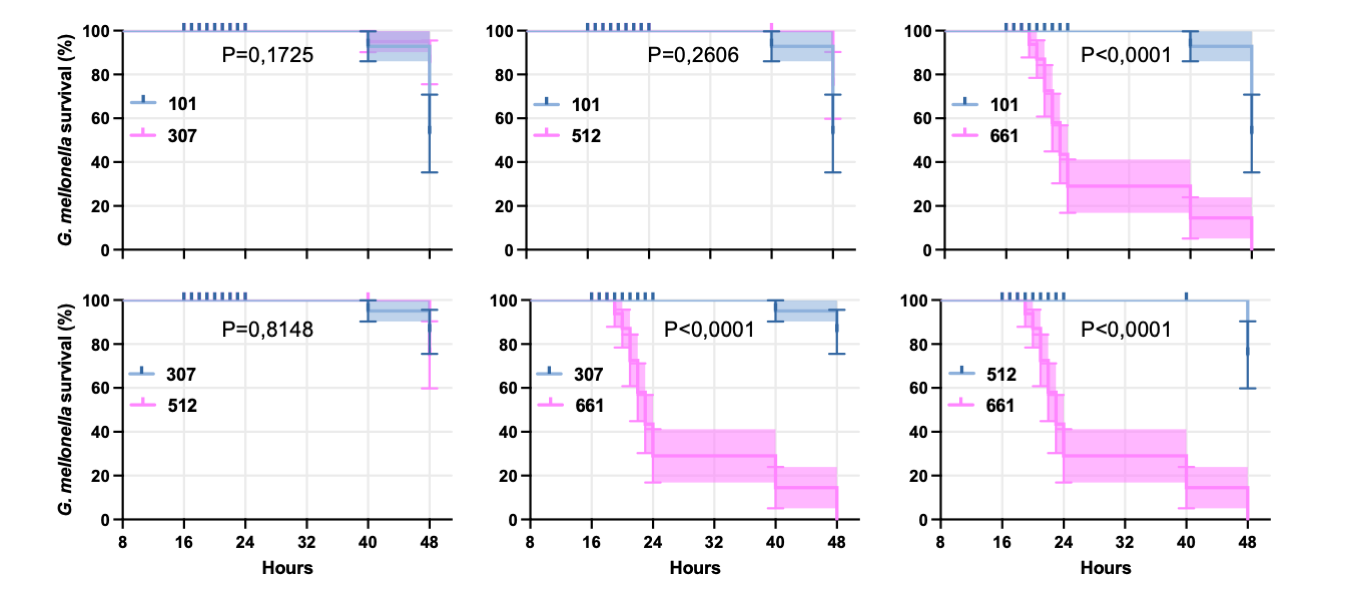
 Comparison of *Galleria mellonella* mortality rates between each sequence type isolates**
